# Correction: Depletion of CD4^+^ CD25^+^ Regulatory T Cells Promotes CCL21-Mediated Antitumor Immunity

**DOI:** 10.1371/journal.pone.0093126

**Published:** 2014-03-27

**Authors:** The PLOS ONE Staff

Some of the work reported in this article was conducted by researchers based at the department of Anatomy, Histology and Embryology, Shanghai Medical College, Fudan University. The authors from Fudan University should have been included in the author list in the article, the authors apologize for this omission.

The author list should be corrected to read as below:

Shuang Zhou1,2☯, Long Chen1☯, Jie Qin1☯, Rilun Li1, Huihong Tao2, Zhiwei Zhen2, Haixia Chen2, Guolin Chen2, Yaoqin Yang2, Binbin Liu3, Zhenjue She1, Cuiping Zhong1, Chunmin Liang1,3*

1 Lab of Tumor Immunology, Department of Anatomy and Histology & Embryology, Shanghai Medical College of Fudan University, Shanghai, China.

2 Department of Histology and Embryology, Tongji University School of Medicine, Shanghai, China.

3 Liver Cancer Institute of Zhongshan Hospital, Shanghai Medical College of Fudan University, Shanghai, China.

☯ These authors equally contributed to this work. * Corresponding author: Chunmin Liang, cmliang@fudan.edu.cn, Principle Investigator of the Lab of Tumor Immunology, Department of Anatomy and Histology & Embryology, Shanghai Medical College, Fudan University, 138 Yixueyuan Road, 200032, Shanghai, China.

The authors from Fudan University declare that no competing interests exist; their contributions to the study are as below:

Conceived and designed the experiments: Chunmin Liang

Performed the animal models: Long Chen

Performed immunostaining experiments: Jie Qin, Rilun Li, Zhenjue She

Performed cell culture assays: Binbin Liu

Analyzed the data: Chunmin Liang, Long Chen

Contributed reagents/materials/analysis tools: Cuiping Zhong, Chunmin Liang

Contributed to the design of the experiments and the writing of the manuscript: Cuiping Zhong

In addition the authors would like to acknowledge Guomin Zhou and Shenglong Ye for their help in providing materials to this work.

The funding statement should also be corrected to acknowledge the grants that supported the authors from Fudan University:

This work was supported by grants from the National Science Foundation of China (No.30871312, No. 31000527), the National Basic Research Program of China (973-program, No.2011CB910700), Shanghai Health Bureau Research Fund for young investigator (to Dr. Shuang Zhou) and Hong Kong Scholar program (No. XJ2011025).
